# Supplementary material for: Insights into the ISG15 transfer cascade by the UBE1L activating enzyme
Source: Nat Commun. 2023 Dec 2;14:7970. doi: 10.1038/s41467-023-43711-3 (PMC10693564; doi:10.1038/s41467-023-43711-3)
Supplement: Supplementary file 1 — Supplementary Information [file 41467_2023_43711_MOESM1_ESM.pdf]

Supplementary Information

Insights into the ISG15 transfer cascade by the UBE1L activating enzyme

Iona Wallace[1], Kheewoong Baek[2], J. Rajan Prabu[2], Ronnald Vollrath[2], Susanne von Gronau[2], Brenda A. Schulman[2]* and Kirby N. Swatek[1,2]*

[1] Medical Research Council Protein Phosphorylation and Ubiquitylation Unit, School of Life Sciences, University of Dundee, Dundee DD1 5EH, UK.

[2] Department of Molecular Machines and Signaling, Max Planck Institute of Biochemistry, Am Klopferspitz 18, 82152 Martinsried, Germany.

* Correspondence to schulman@biochem.mpg.de; kswatek001@dundee.ac.uk

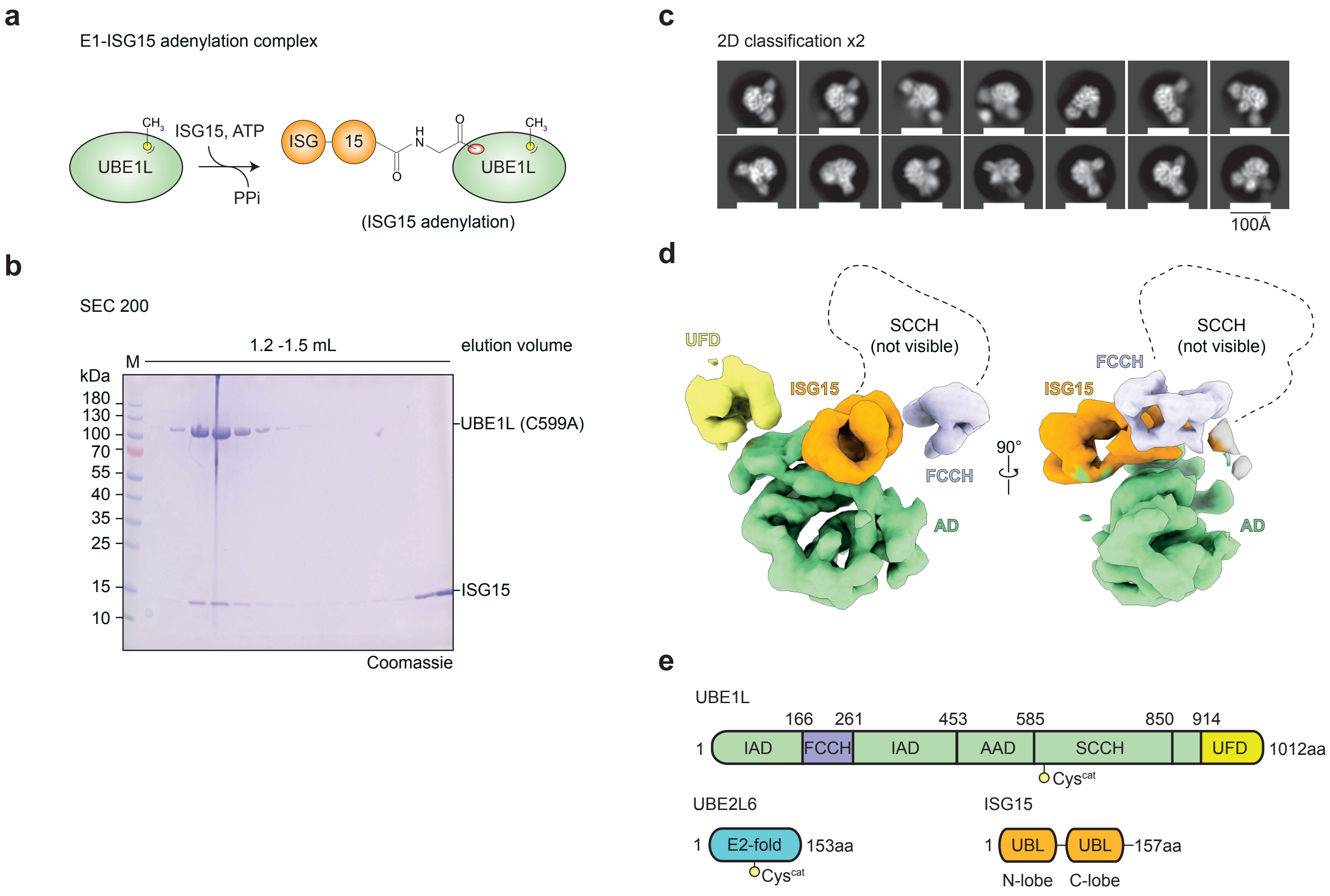

Supplementary Figure 1. Cryo-EM analysis of the UBE1L-ISG15 complex.
a, Schematic of UBE1L-ISG15 complex formation. Mutation of the UBE1L active site cysteine stabilizes the adenylated ISG15 intermediate by preventing downstream thioester bond formation (also see **Fig. 1**). **b**, Analytical size exclusion chromatography (SEC) of the UBE1L-ISG15 complex. Fractions were resolved by SDS-PAGE and visualized with Coomassie stain. Formation of the UBE1L-ISG15 complex was performed independently in duplicate. **c**, Representative 2-dimensional (2D) classes used for reconstruction of the UBE1L-ISG15 cryo-EM map. **d**, Cryo-EM density representing the UBE1L-ISG15 complex, in which ISG15 is bound to the adenylation domain (AD). The SCCH domain is not visible, likely due to its flexibility. **e**, Colouring scheme of UBE1L domains, UBE2L6, and ISG15. Adenylation domain (includes IAD: inactive adenylation domain and AAD: active adenylation domain); FCCH: first catalytic cysteine half-domain; SCCH: second catalytic cysteine half-domain; UFD: ubiquitin fold domain; E2-fold: E2 conjugating enzyme fold; Ubl: ubiquitin-like protein fold. Source data are provided in the Source Data file.

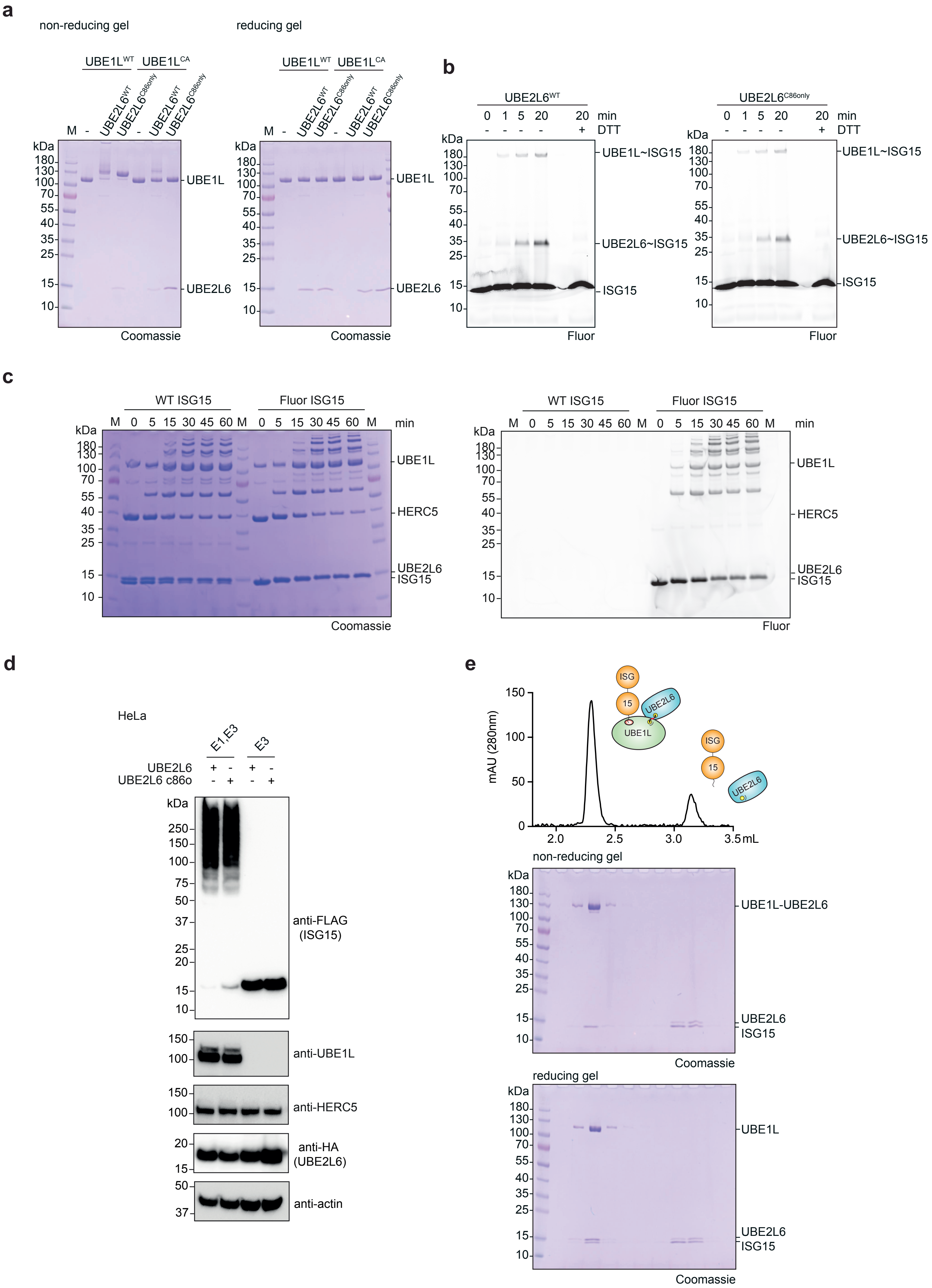
a
non-reducing gel
reducing gel
UBE1L^WT
UBE1L^CA
UBE2L6^WT
UBE2L6^C86only
M
kDa
180
130
100
70
55
40
35
25
15
10
UBE1L
UBE2L6
Coomassie
b
UBE2L6^WT
UBE2L6^C86only
0 1 5 20 20 min
- - - - + DTT
UBE1L~ISG15
UBE2L6~ISG15
ISG15
Fluor
c
WT ISG15
Fluor ISG15
M 0 5 15 30 45 60 M 0 5 15 30 45 60 M min
UBE1L
HERC5
UBE2L6
ISG15
Coomassie
Fluor
d
HeLa
E1,E3
E3
UBE2L6 + - + -
UBE2L6 c86o - + - +
kDa
250
150
100
75
50
37
25
20
15
10
anti-FLAG (ISG15)
anti-UBE1L
anti-HERC5
anti-HA (UBE2L6)
anti-actin
e
mAU (280nm)
150
100
50
0
2.0
2.5
3.0
3.5 mL
ISG
15
UBE2L6
UBE1L
non-reducing gel
UBE1L-UBE2L6
UBE2L6
ISG15
Coomassie
reducing gel
UBE1L
UBE2L6
ISG15
Coomassie

Supplementary Figure 2. Formation of the ISG15 E1-E2 disulfide-linked complex.

a, UBE1L-UBE2L6 disulfide bond formation. Non-reducing and reducing SDS-PAGE gels demonstrate site-specific disulfide bond formation between the catalytic cysteines of wild-type UBE1L (UBE1L^{WT}) and UBE2L6 Cys86-only (UBE2L6^{C86only}). UBE2L6^{C86only} is a mutant in which all cysteine residues, except the active site cysteine, are mutated to serine residues. UBE1L^{CA} contains a single Ala mutation at the active site (Cys599Ala). Reactions were separated by SDS-PAGE and visualized with Coomassie stain. **b**, Comparison of ISG15 E2 charging with wild-type UBE2L6 (UBE2L6^{WT}) and UBE2L6^{C86only}. Reactions were separated by SDS-PAGE and visualized with fluorescently labelled ISG15 at the indicated time points. **c,** In vitro ISG15 assembly reactions comparing unlabelled ISG15 (WT ISG15) and fluorescent ISG15 (Fluor ISG15), confirming the ISG15 machinery is not affected by fluorescent labelling of ISG15. Reactions were visualised with Coomassie stain and fluorescent imaging. **d**, Comparison of UBE2L6^{WT} and UBE2L6^{C86only} activity in cells. HeLa cells were transfected with UBE2L6^{WT} or UBE2L6^{C86only}, and additional components of ISG15 machinery (FLAG-ISG15, UBE1L, HERC5). As a control, identical transfection assays were performed without UBE1L. The accumulation of ISGylated substrates was monitored with an anti-FLAG antibody and the expression of the ISG15 conjugation machinery was visualised using the indicated antibodies. **e**, Analytical size exclusion profile of the ISG15 UBE1L-UBE2L6 complex (top). Fractions were separated with non-reducing (middle) and reducing (bottom) SDS-PAGE gels and visualized with Coomassie stain. Assays from **a**, **b**, and **d** were performed independently in triplicate. Assays in **c** and **e** were performed independently in duplicate. Source data are provided in the Source Data file.

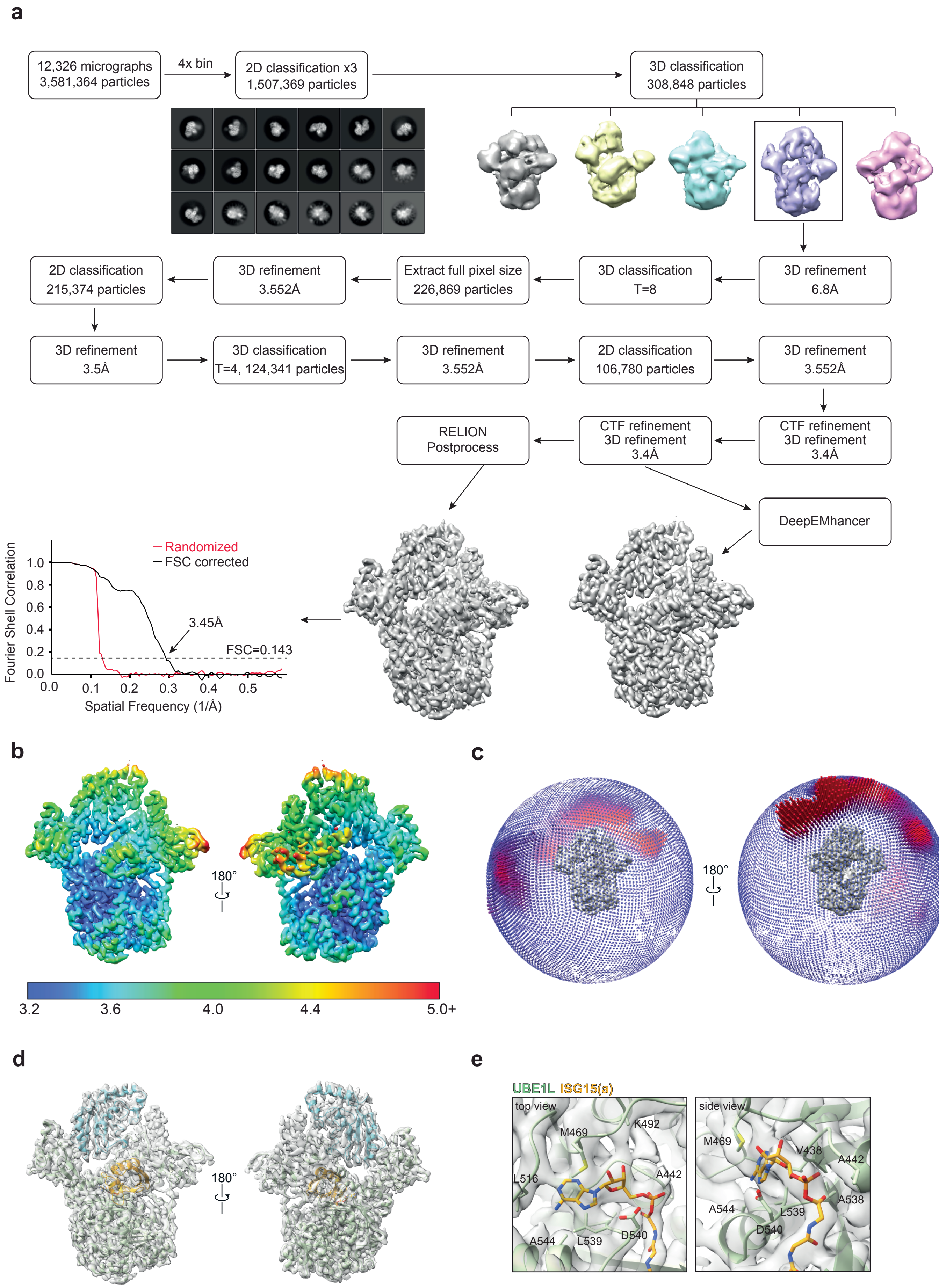

a
12,326 micrographs
3,581,364 particles
4x bin
2D classification x3
1,507,369 particles
3D classification
308,848 particles
3D refinement
6.8Å
3D classification
T=8
Extract full pixel size
226,869 particles
3D refinement
3.552Å
2D classification
215,374 particles
3D refinement
3.5Å
3D classification
T=4, 124,341 particles
3D refinement
3.552Å
2D classification
106,780 particles
3D refinement
3.552Å
CTF refinement
3D refinement
3.4Å
CTF refinement
3D refinement
3.4Å
RELION
Postprocess
DeepEMhancer
Randomized
FSC corrected
3.45Å
FSC=0.143
Fourier Shell Correlation
Spatial Frequency (1/Å)
b
180°
3.2
3.6
4.0
4.4
5.0+
c
180°
d
180°
e
UBE1L ISG15(a)
top view
side view
M469
K492
L516
A442
A544
L539
D540
V438
A538

Supplementary Figure 3. Cryo-EM image processing flow chart of the ISG15 C-lobe UBE1L-UBE2L6 complex.

a, Cryo-EM image reconstruction flow chart. The following scheme generated a cryo-EM map with an overall resolution of 3.45 Å. Insets show the two-dimensional (2D) and three-dimensional (3D) classes used for further processing. For comparison, post-processing was performed using both RELION and DeepEMhancer. DeepEMhancer generated a comparable, but noticeably sharper cryo-EM map. The Fourier shell correlation (FSC) threshold for resolution estimation was set to 0.143, as previously described[1]. **b**, Local resolution of the DeepEMhancer cryo-EM map. The highest resolution can be seen within the E1 adenylation domain and E1-ISG15 contacts. **c**, Angular distribution of the final cryo-EM map. **d**, Overall map to model fit for ISG15 C-lobe UBE1L-UBE2L6 complex. **e**, Map to model fit for adenylated ISG15 (ISG15(a)) within the adenylation domain active site of UBE1L.

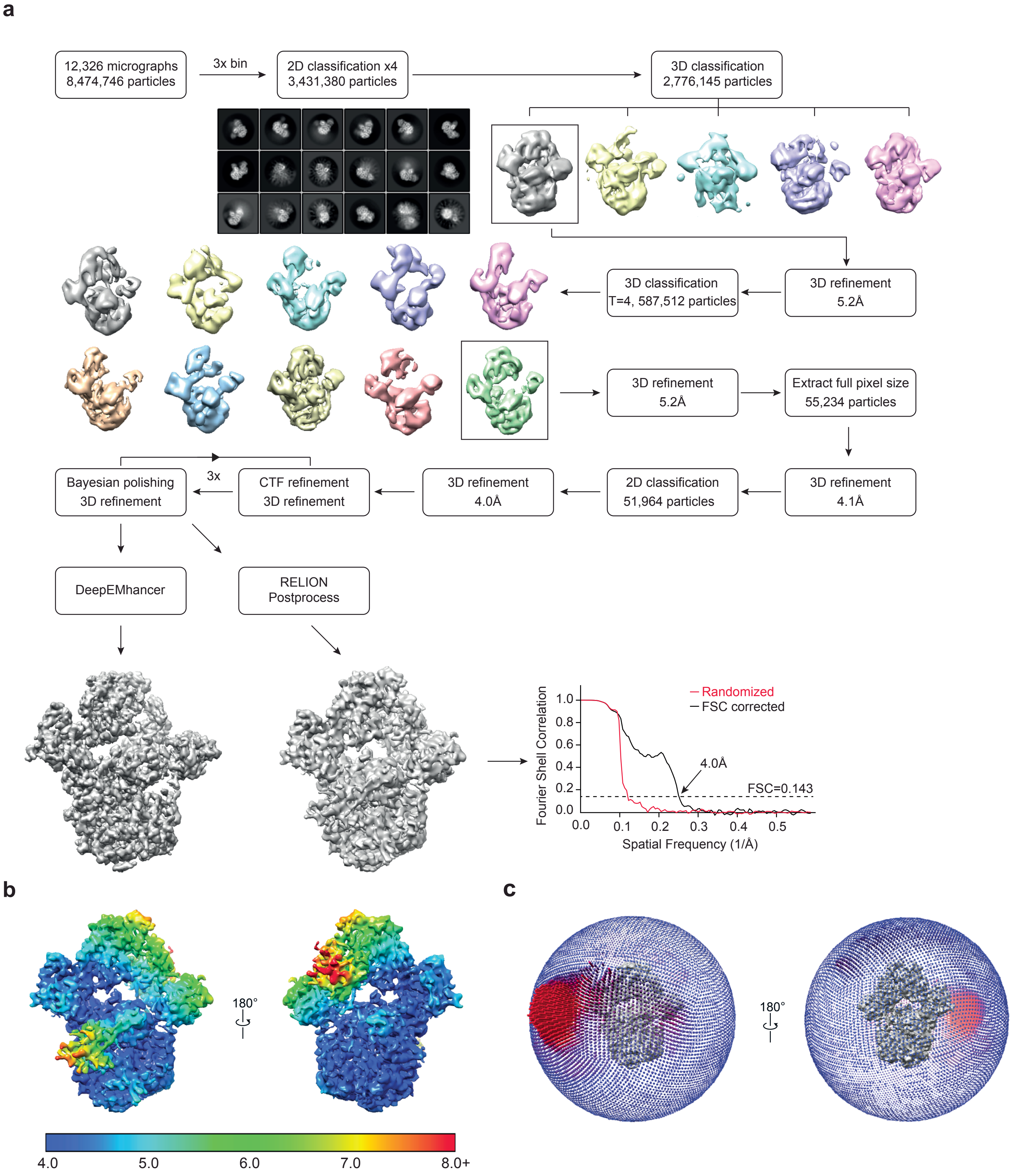

a
12,326 micrographs
8,474,746 particles
3x bin
2D classification x4
3,431,380 particles
3D classification
2,776,145 particles
3D classification
T=4, 587,512 particles
3D refinement
5.2Å
3D refinement
5.2Å
Extract full pixel size
55,234 particles
3D refinement
4.1Å
2D classification
51,964 particles
3D refinement
4.0Å
CTF refinement
3D refinement
3x
Bayesian polishing
3D refinement
DeepEMhancer
RELION
Postprocess
Randomized
FSC corrected
Fourier Shell Correlation
1.0
0.8
0.6
0.4
0.2
0.0
4.0Å
FSC=0.143
0.0
0.1
0.2
0.3
0.4
0.5
Spatial Frequency (1/Å)
b
180°
4.0
5.0
6.0
7.0
8.0+
c
180°

Supplementary Figure 4. Cryo-EM image processing flow chart of the full length ISG15 UBE1L-UBE2L6 complex.

a, Cryo-EM image reconstruction flow chart. The following scheme generated a cryo-EM map with an overall resolution of 4.0 Å. Insets show the two-dimensional (2D) and three-dimensional (3D) classes used for further processing. For comparison, post-processing was performed using both RELION and DeepEMhancer. DeepEMhancer generated a comparable, but noticeably sharper cryo-EM map (also see **Fig. 2d**). The Fourier shell correlation (FSC) threshold for resolution estimation was set to 0.143, as previously described[1]. **b**, Local resolution of the cryo-EM map. The highest resolution can be seen within the E1 adenylation domain and E1-ISG15 contacts. **c**, Angular distribution of the final cryo-EM map.

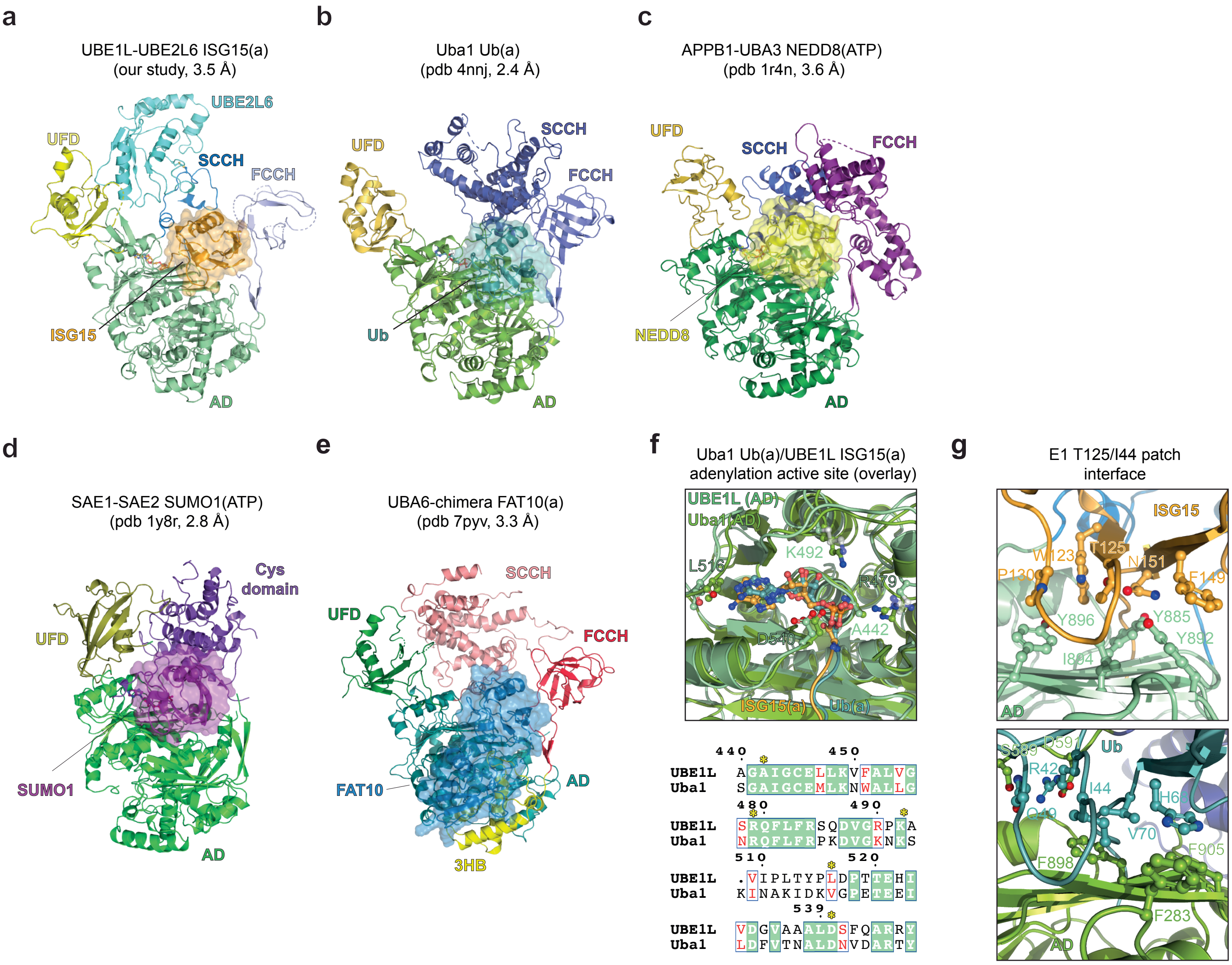
a
UBE1L-UBE2L6 ISG15(a)
(our study, 3.5 Å)
UBE2L6
UFD
SCCH
FCCH
ISG15
AD
b
Uba1 Ub(a)
(pdb 4nnj, 2.4 Å)
SCCH
UFD
FCCH
Ub
AD
c
APPB1-UBA3 NEDD8(ATP)
(pdb 1r4n, 3.6 Å)
UFD
SCCH
FCCH
NEDD8
AD
d
SAE1-SAE2 SUMO1(ATP)
(pdb 1y8r, 2.8 Å)
Cys
domain
UFD
SUMO1
AD
e
UBA6-chimera FAT10(a)
(pdb 7pyv, 3.3 Å)
SCCH
UFD
FCCH
FAT10
AD
3HB
f
Uba1 Ub(a)/UBE1L ISG15(a)
adenylation active site (overlay)
UBE1L (AD)
Uba1 (AD)
K492
L516
R479
D540
A442
ISG15(a)
Ub(a)
440 450
UBE1L AGAIGCELLKVFALVG
Uba1 SGAIGCEMLKNWALLG
480 490
UBE1L SRQFLFRSQDVGRPKA
Uba1 NRQFLFRPKDVGKNKS
510 520
UBE1L .VIPLTYPLDPTTEHI
Uba1 KINAKIDKVGPETEEI
539
UBE1L VDGVAAALDSFQARRY
Uba1 LDFVTNALDNVDARTY
g
E1 T125/I44 patch
interface
ISG15
W123
T125
P130
N151
F149
Y896
Y885
Y892
I894
AD
D591
Ub
R42
I44
V70
F898
F905
F283
AD

Supplementary Figure 5. Structural comparison of E1-ubiquitin and E1-ubiquitin-like protein complexes.

a, Structural overview of adenylated ISG15 (ISG15(a)) bound to disulfide-linked UBE1L-UBE2L6 (also see **Fig. 2**). **b**, Structure of adenylated ubiquitin (Ub(a)) bound to Uba1 (pdb 4nnj)[2]. Uba1 has a similar overall domain architecture to UBE1L. **c**, Structure of heterodimeric NEDD8 E1 (APPB1-UBA3) bound to NEDD8 (pdb 1r4n)[3]. Compared to the FCCH domains of Uba1 and UBE1L, the FCCH domain of NEDD8 E1 is large and helical. **D**, Structure of heterodimeric SUMO E1 (SAE1-SAE2) bound to SUMO1 (pdb 1y8r)[4]. A unique feature of the SUMO E1 is a small-disordered FCCH domain. **e**, Structure of FAT10 E1 chimera (UBA6chimera; the SCCH domain of UBA6 was replaced with the UBA1 SCCH domain) bound to FAT10 (pdb 7pyv)[5]. The UBA6 3-helix bundle (3HB) contacts the N-terminal ubiquitin-like domain of FAT10. **f,** Top, overlay of adenylated ISG15 C-terminus within the adenylation domain (AD) active site of UBE1L, and adenylated ubiquitin C-terminus within the AD active site of Uba1 (pdb 4nnj)[2]. UBE1L side chain residues shown in transparent grey indicate regions with missing cryo-EM density, where side chains have been modelled in a similar orientation to Uba1 residues. Bottom, sequence alignment of UBE1L adenylation domain residues to the equivalent residues in Uba1. Yellow asterisk represents Uba1 residues which interact with the adenylated C-terminus of ubiquitin[2] and are conserved in UBE1L. **g**, Close-up view of UBE1L and Uba1 Ubl/Ub interactions with the adenylation domain. The Ile44 patch of Ub contacts Uba1 hydrophobic residues (Phe283, Phe898, Phe905). Nearby Ub residues (e.g., Arg42) also contact Uba1. Comparison to the corresponding UBE1L-ISG15 interface (T125 patch) reveals unique hydrogen bonds and hydrophobic interactions.

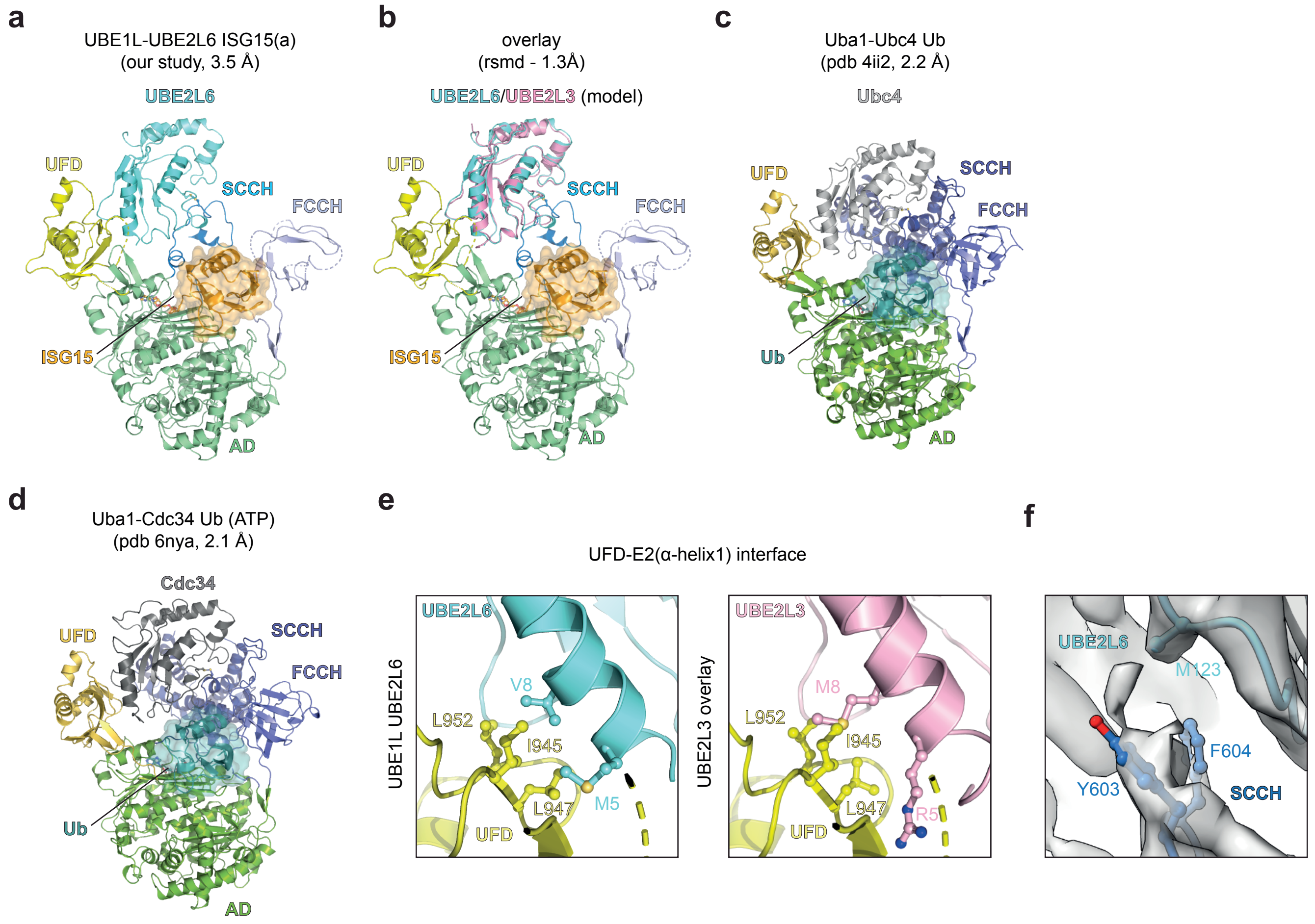

Supplementary Figure 6. Structural comparison of E1-E2 disulfide-linked complexes and analysis of UBE2L6 specificity.
a, Overview of adenylated ISG15 bound to the UBE1L-UBE2L6 disulfide-linked complex (also see **Fig. 2**). **b**, Model of adenylated ISG15 bound to the UBE1L-UBE2L3 complex. UBE2L3 was superimposed onto the UBE2L6 structure from **a** (UBE2L3 pdb 6djx; Cα rsmd = 1.3 Å)[6]. **c**, Structure of ubiquitin (Ub) bound to the Uba1-Ubc4 complex (pdb 4ii2)[7]. Like UBE1L, the UFD and SCCH domains position the E2 enzyme. **d**, Structure of Ub bound to the Uba1-Cdc34 complex (pdb 6nya)[8]. The overall structure resembles complexes in **a** and **c.** **e**, Close-up view comparing the UBE1L-UBE2L6 and UBE1L-UBE2L3 (model) interactions. **f**, Cryo-EM density surrounding UBE2L6 Met123. Hydrophobic UBE1L residues within the SCCH domain (Tyr603, Phe604) are located in close proximity to UBE2L6 Met123, thereby forming an E1-E2 interface.

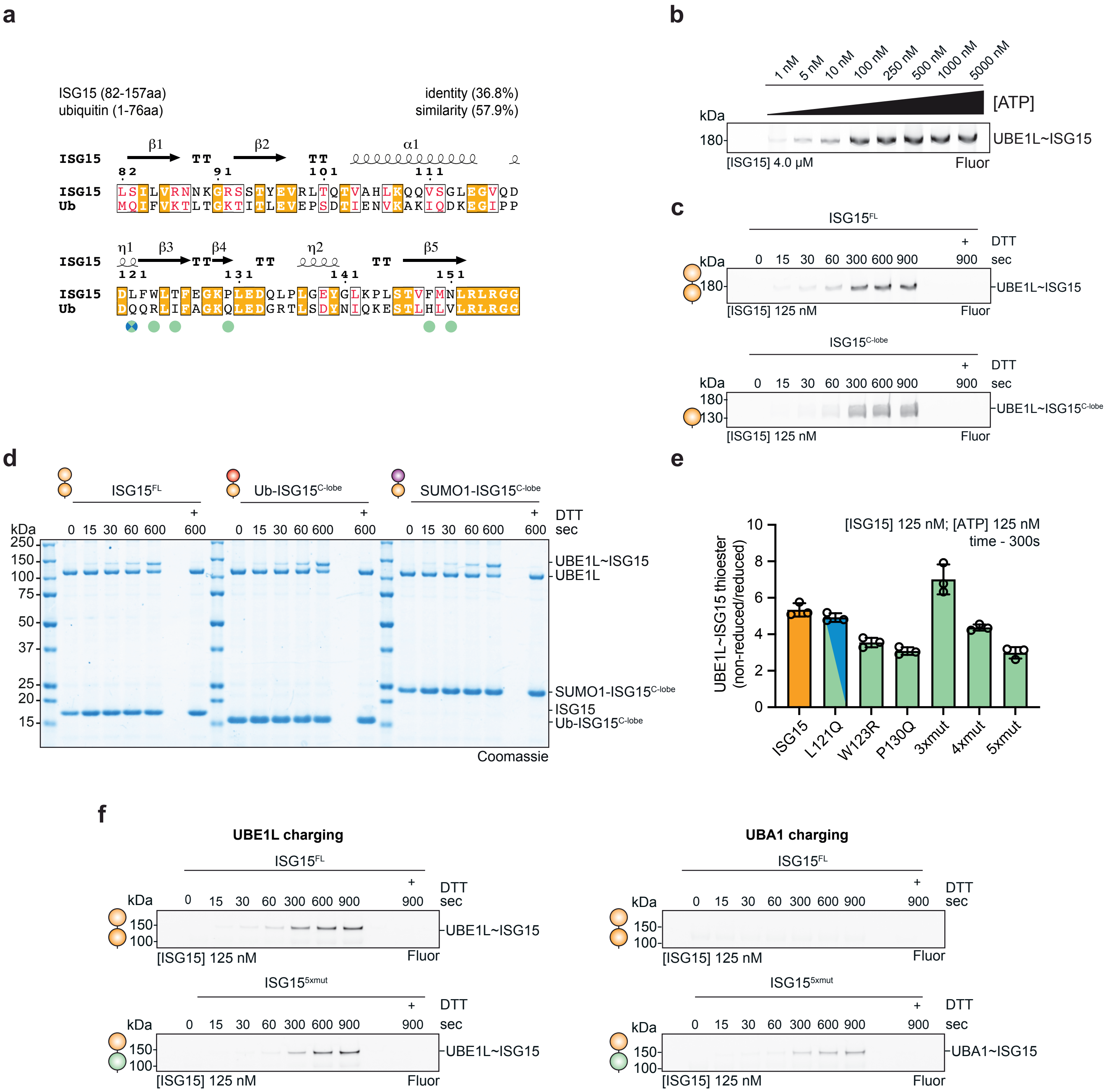
a
ISG15 (82-157aa)
ubiquitin (1-76aa)
identity (36.8%)
similarity (57.9%)
ISG15
β1 TT β2 TT α1
82 91 101 111
ISG15 LSILVRNNKGRSSTYEVRLTQTVAHLKQQVSGLEGVQD
Ub MQIFVKTLTGKTITLEVEPSDTIENVKAKIQDKEGIPP
ISG15
η1 β3 TT β4 TT η2 TT β5
121 131 141 151
ISG15 DLFWLTFEGKPLEDQLPLGEYGLKPLSTVFMNLRLRGG
Ub DQQRLIFAGKQLEDGRTLSDYNIQKESTLHLVLRLRGG
b
1 nM
5 nM
10 nM
100 nM
250 nM
500 nM
1000 nM
5000 nM
[ATP]
kDa
180
UBE1L~ISG15
[ISG15] 4.0 µM
Fluor
c
ISG15FL
+ DTT
kDa 0 15 30 60 300 600 900 900 sec
180
UBE1L~ISG15
[ISG15] 125 nM
Fluor
ISG15C-lobe
+ DTT
kDa 0 15 30 60 300 600 900 900 sec
180
130
UBE1L~ISG15C-lobe
[ISG15] 125 nM
Fluor
d
ISG15FL
Ub-ISG15C-lobe
SUMO1-ISG15C-lobe
+ + + DTT
kDa 0 15 30 60 600 600 0 15 30 60 600 600 0 15 30 60 600 600 sec
250
150
100
75
50
37
25
20
15
UBE1L~ISG15
UBE1L
SUMO1-ISG15C-lobe
ISG15
Ub-ISG15C-lobe
Coomassie
e
[ISG15] 125 nM; [ATP] 125 nM
time - 300s
UBE1L~ISG15 thioester
(non-reduced/reduced)
10
8
6
4
2
0
ISG15
L121Q
W123R
P130Q
3xmut
4xmut
5xmut
f
UBE1L charging
ISG15FL
+ DTT
kDa 0 15 30 60 300 600 900 900 sec
150
100
UBE1L~ISG15
[ISG15] 125 nM
Fluor
ISG155xmut
+ DTT
kDa 0 15 30 60 300 600 900 900 sec
150
100
UBE1L~ISG15
[ISG15] 125 nM
Fluor
UBA1 charging
ISG15FL
+ DTT
kDa 0 15 30 60 300 600 900 900 sec
150
100
[ISG15] 125 nM
Fluor
ISG155xmut
+ DTT
kDa 0 15 30 60 300 600 900 900 sec
150
100
UBA1~ISG15
[ISG15] 125 nM
Fluor

Supplementary Figure 7. Biochemical characterization of UBE1L charging.
a, Sequence alignment of the ISG15 C-terminal ubiquitin-like domain to ubiquitin (Ub). Sequence identity and similarity are indicated. Coloured circles denote the residues which contact the UBE1L domains (green – adenylation domain; crossover loop – blue-green mix). Sequence alignments were performed using T-Coffee[9] and ESPript[10]. **b**, ATP-dependence of UBE1L charging using fluorescent ISG15. Reactions were performed with 4 μM ISG15 and 2 μM UBE1L at the indicated ATP concentrations. Reactions were separated by SDS-PAGE and visualised with fluorescent imaging. **c,** UBE1L charging reactions with fluorescent full-length ISG15 ($ISG15^{FL}$) and fluorescent ISG15 C-lobe ($ISG15^{C\text{-}lobe}$) at a rate-limiting concentration of ISG15. Reactions were visualised as in **b. d,** Full gel images from **Fig. 4d**. **e,** Quantification of UBE1L charging with a rate-limiting concentrations of wild-type ISG15 and ISG15 mutants. ISG15 point mutations were in UBE1L contact sites and mutated residues were swapped with the analogous residues of ubiquitin (also see **a** and **Fig. 4a**). Error values represent s.d. from the mean (n=3 independent experiments). Samples derive from same experiment and gels were processed in parallel. **f,** UBE1L and UBA1 charging reactions comparing full-length ISG15 ($ISG15^{FL}$) and $ISG15^{5xmut}$ (W123R/T125I/P130Q/F149H/N151V). Reactions were performed as in **c**. Experiments were performed independently in triplicate. Source data are provided in the Source Data file.

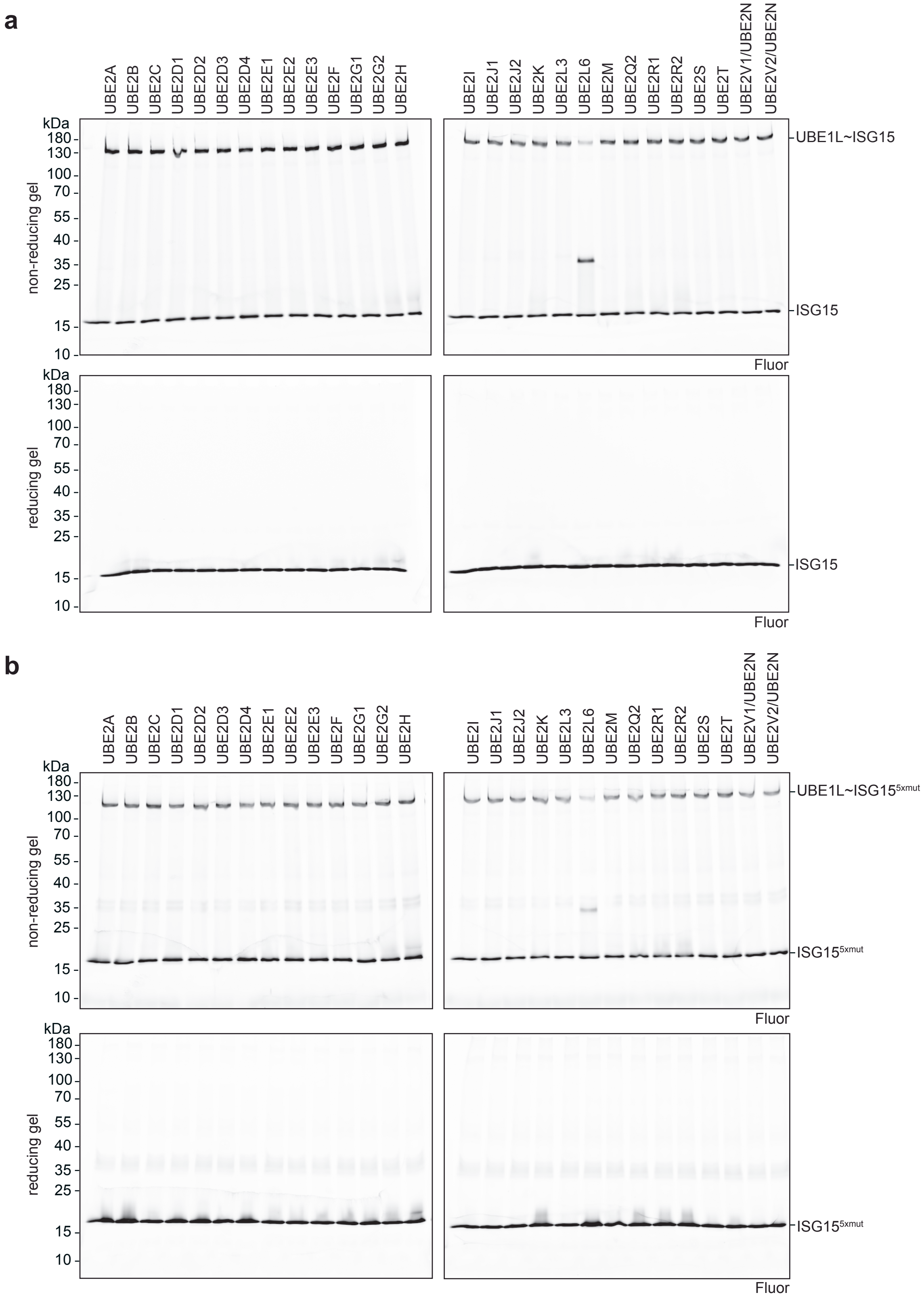
a
UBE2A
UBE2B
UBE2C
UBE2D1
UBE2D2
UBE2D3
UBE2D4
UBE2E1
UBE2E2
UBE2E3
UBE2F
UBE2G1
UBE2G2
UBE2H
UBE2I
UBE2J1
UBE2J2
UBE2K
UBE2L3
UBE2L6
UBE2M
UBE2Q2
UBE2R1
UBE2R2
UBE2S
UBE2T
UBE2V1/UBE2N
UBE2V2/UBE2N
kDa
180
130
100
70
55
40
35
25
15
10
non-reducing gel
reducing gel
UBE1L~ISG15
ISG15
Fluor
b
UBE1L~ISG15^5xmut
ISG15^5xmut
Fluor

Supplementary Figure 8. Biochemical characterization of UBE2L6 charging.
a, Comprehensive analysis of UBE1L-mediated E2 charging with ISG15. As expected, UBE2L6 is the only E2 enzyme that forms a thioester bond with ISG15. Reactions were separated with non-reducing (top) and reducing (bottom) SDS-PAGE gels and visualized with fluorescent imaging. **b**, Comprehensive analysis of UBE1L-mediated E2 charging with $ISG15^{5xmut}$. Similar to ISG15, UBE2L6 is the only E2 that forms a thioester bond with $ISG15^{5xmut}$. Reactions were separated and visualized as in **a**. Experiments were performed independently in triplicate. Source data are provided in the Source Data file.

a

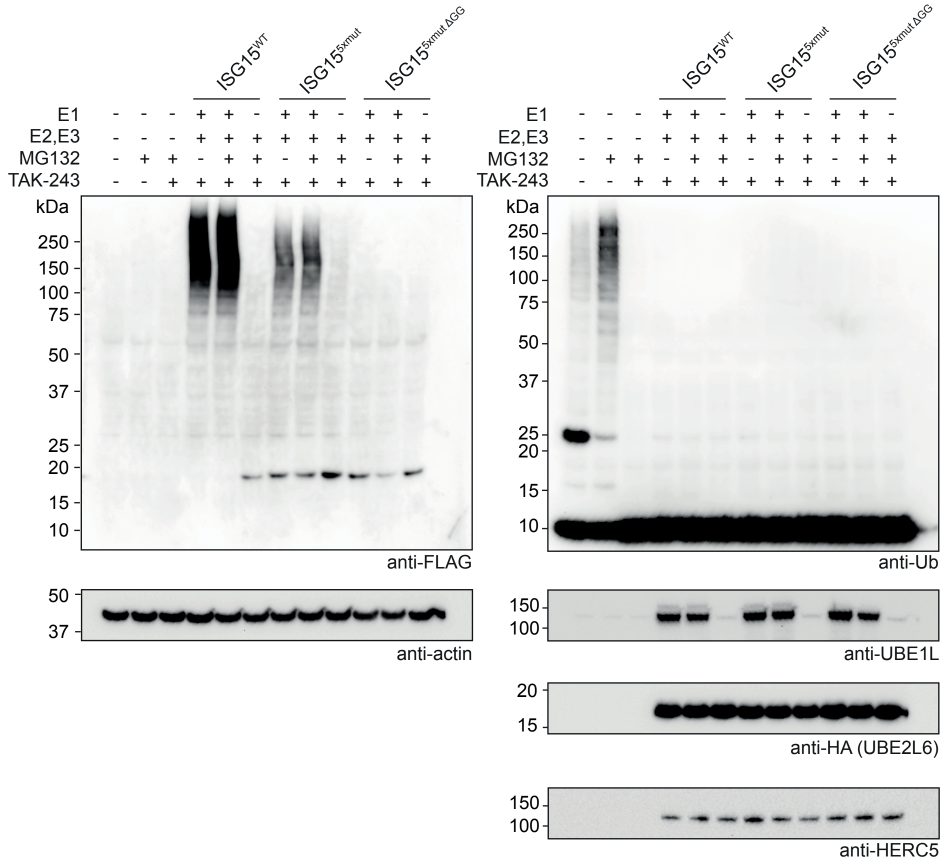

Supplementary Figure 9. Assessing the ability of ubiquitylised ISG15 to function with the ISG15 enzyme cascade. a, HeLa cells were transfected with the ISG15 enzyme cascade (E1-E2-E3) and either full-length ISG15 (ISG15^{FL}), ISG15^{5xmut} (W123R/T125I/P130Q/F149H/N151V) or a non-conjugatable version of ISG15^{5xmut} (ISG15$^{5xmut\ \Delta GG}$). As a control, identical transfection assays were performed without UBE1L. Transfected cells were subsequently treated with the UBA1 inhibitor TAK-243 or TAK-243 and MG132. The accumulation of ISGylated substrates was monitored with an anti-FLAG antibody and co-expression of the ISG15 machinery was confirmed using the indicated antibodies. Actin and ubiquitin western blots were used to control for loading and inhibitor treatment, respectively. Experiments were performed independently in triplicate. Source data are provided in the Source Data file.

Supplementary Figure 10

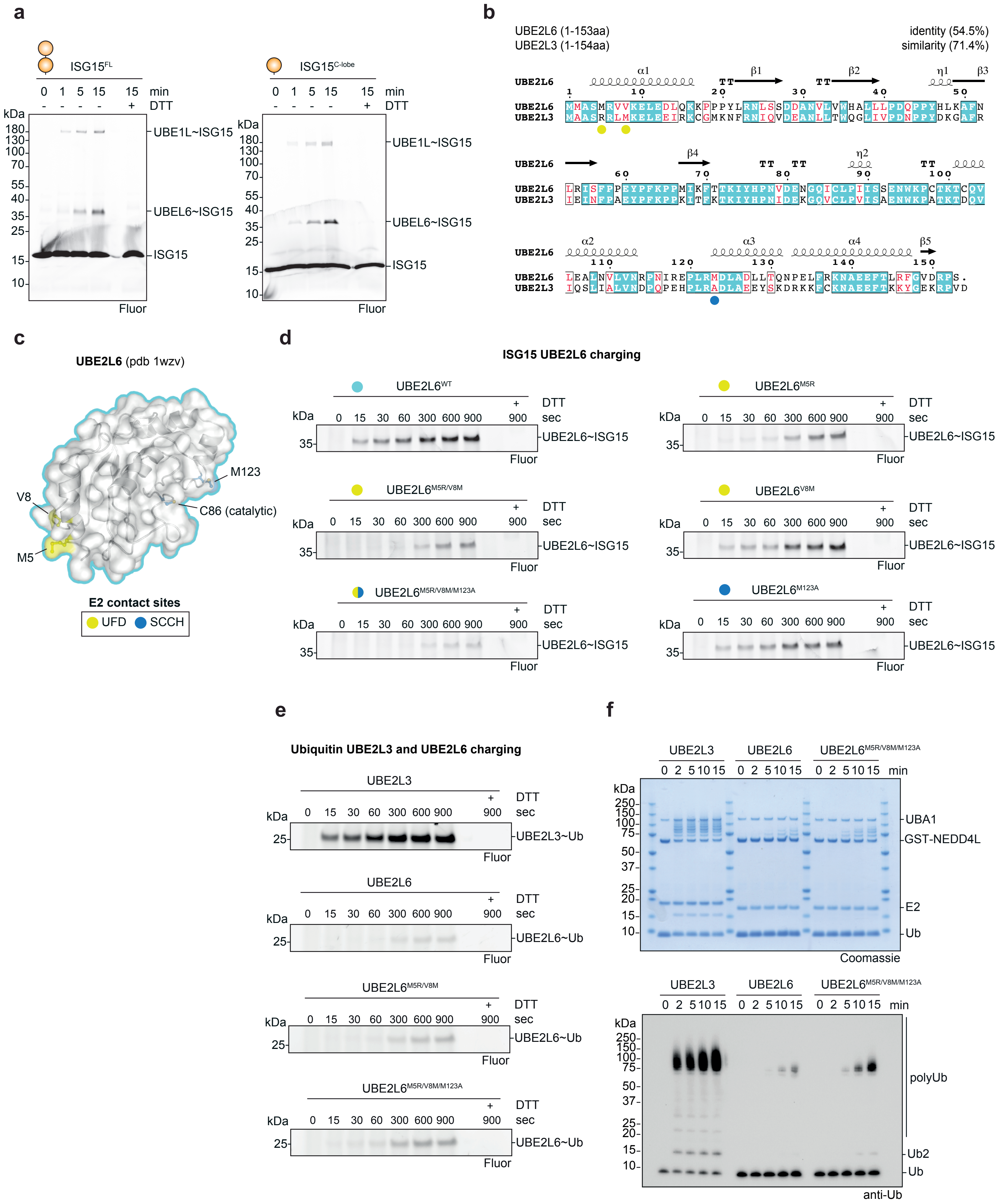

Supplementary Figure 10. Characterisation of UBE2L6 specificity.
a, UBE2L6 charging assays comparing full-length ISG15 ($ISG15^{FL}$) and the ISG15 C-terminal ubiquitin-like fold ($ISG15^{C\text{-}lobe}$). Reactions were separated by SDS-PAGE and visualized with fluorescent imaging. **b**, Sequence alignment of UBE2L6 and UBE2L3. Sequence identity and similarity are indicated. Coloured circles denote the residues which contact UBE1L domains (yellow – ubiquitin fold domain (UFD), blue – second catalytic cysteine half-domain (SCCH). Sequence alignments were performed using T-Coffee[9] and ESPript[10]. **c**, Structure of UBE2L6 highlighting residues that contact UBE1L in the cryo-EM structure (pdb 1wzv)[11]. Residues are shown in ball-and-stick representation under a semi-transparent surface. Residues contacting the UFD (yellow) and SCCH (blue) are shown. Structural analysis identified α-helix1 residues (Met5, Val8) as the primary contact sites with UBE1L (also see **Fig. 2** and **3**). **d,** UBE2L6 mutant charging assays with $ISG15^{FL}$. Reactions were separated by SDS-PAGE and visualized with fluorescent imaging at the indicated time points. **e,** UBA1-mediated charging of UBE2L3, UBE2L6 and UBE2L6 mutants with ubiquitin. **f,** In vitro ubiquitin assembly reactions comparing UBE2L3, UBE2L6 and $UBE2L6^{M5R/V8M/M123A}$ activity with the ubiquitin E3 ligase NEDD4L. Reactions were visualised with Coomassie stain and anti-ubiquitin (Ub) western blots. Experiments were performed independently in triplicate. Source data are provided in the Source Data file.

Cryo-EM data collection, refinement and validation statistics

Complex	**UBE1L-UBE2L6 with ISG15**	
ISG15 density	full-length	C-lobe (only)
Accession	EMDB-18589	EMDB-16891 (PDB:8OIF)
**Data collection and processing**		
Microscope	Krios	Krios
Magnification	105,000	105,000
Voltage (kV)	300	300
Electron exposure (e–/Å^2)	61.5	61.5
Defocus range (μm)	-1.2 ~ -3.6	-1.2 ~ -3.6
Pixel size (Å)	0.8512	0.8512
Symmetry imposed	C1	C1
Initial particle images (no.)	8,474,746	3,581,364
Final particle images (no.)	51,964	106,780
Map resolution (Å)	4.0	3.45
FSC threshold	(0.143)	(0.143)
Map resolution range (Å)	3.8 ~ 8.2	3.2 ~ 5.7
**Refinement**		
Initial model used (PDB:code)		6FFA(B) 1WZV UBE1L (model)
Model resolution (Å)		3.7
FSC threshold		(0.143)
Map sharpening *B* factor (Å^2)		-90
Model composition		
Non-hydrogen atoms		6589
Protein residues		934
Ligands		1(AMP)
B factors (Å^2)		
Protein		55.10
Ligand		50.59
R.M.S. deviations		
Bond lengths (Å)		0.003
Bond angles (°)		0.595
Validation		
MolProbity score		1.77
Clashscore		6.05
Poor rotamers (%)		0
Ramachandran plot		
Favored (%)		93.2
Allowed (%)		6.8
Disallowed (%)		0

Supplementary Table 1. Cryo-EM data collection, refinement and validation statistics

primer name	sequence
UBE2L6_M5R_*forward*	GCGAGCAGACGAGTGGTGAAGGAGCTGGAGGATC
UBE2L6_M5R_*reverse*	CACTCGTCTGCTCGCCATCATTCCACCGGTTTG
UBE2L6_V8M_*forward*	CGAGTGATGAAGGAGCTGGAGGATCTTCAGAAGAAGCC
UBE2L6_V8M_*reverse*	CTCCTTCATCACTCGCATGCTCGCCATCATTCC
UBE2L6_M5R_V8M_*forward*	GCGAGCAGACGAGTGATGAAGGAGCTGGAGGATCTTCAGAAGAAGCC
UBE2L6_M5R_V8M_*reverse*	CTCCTTCATCACTCGTCTGCTCGCCATCATTCCACCGGTTTG
UBE2L6_M123A_*forward*	CTGCGGGCGGACCTCGCTGACCTGCTGACACAG
UBE2L6_M123A_*reverse*	GAGGTCCGCCCGCAGGGGCTCCCTGATATTCG
ISG15_L121Q_*forward*	GACGACCAGTTCTGGCTGACCTTCGAGGGGAAG
ISG15_L121Q_*reverse*	CCAGAACTGGTCGTCCTGCACACCCTCCAGC
ISG15_T125I_*forward*	CTGGCTGATCTTCGAGGGGAAGCCCCTGGAG
ISG15_T125I_*reverse*	CTCGAAGATCAGCCAGAACAGGTCGTCCTGCAC
ISG15_F149H_*forward*	CACCGTGCACATGAATCTGCGCCTGCGGGGAGGC
ISG15_F149H_*reverse*	GATTCATGTGCACGGTGCTCAGGGGCTTGAGG
ISG15_N151V_*forward*	GTTCATGGTCCTGCGCCTGCGGGGAGGC
ISG15_N151V_*reverse*	GCGCAGGACCATGAACACGGTGCTCAGGGGCTTGAG
ISG15_W123R_*forward*	CTGTTCAGACTGACCTTCGAGGGGAAGCCCC
ISG15_W123R_*reverse*	GGTCAGTCTGAACAGGTCGTCCTGCACACCCTC
ISG15_F149H_N151V_*forward*	GAGCACCGTGCACATGGTCCTGCGCCTGCGGGGAGGC
ISG15_F149H_N151V_*reverse*	CAGGCGCAGGACCATGTGCACGGTGCTCAGGGGCTTGAGGCC
ISG15_P130Q_*forward*	GGGAAGCAACTGGAGGACCAGCTCCCGCTG
ISG15_P130Q_*reverse*	CTCCAGTTGCTTCCCCTCGAAGGTCAGCCAGAAC
ISG15_P130Q_(with3xmut)_*reverse*	CTCCAGTTGCTTCCCCTCGAAGATCAGCCAGAACAG
ISG15_W123R_(with4xmut)_*forward*	CTGTTCAGACTGATCTTCGAGGGGAAGCAACTGGAG
ISG15_W123R_(with4xmut)_reverse	GATCAGTCTGAACAGGTCGTCCTGCACACCCTC
UBE1L_C599A_*forward*	CCCCCTACCCTGTCGCTACCGTGCGGTACT
UBE1L_C599A_reverse	AGTACCGCACGGTAGCGACAGGGTAGGGGG
PLpro_C111A_*forward*	GATAACAACGCATATCTGGCTACCGCGCTGCTGACTCTG
PLpro_C111A_*reverse*	GCCAGATATGCGTTGTTATCGGCCCACTTGATAGAGGTCAGAC

Supplementary Table 2. Primers used in this study.

Supplementary References

1. Rosenthal, P. B. & Henderson, R. Optimal Determination of Particle Orientation, Absolute Hand, and Contrast Loss in Single-particle Electron Cryomicroscopy. *J Mol Biol* **333**, 721–745 (2003).

2. Schäfer, A., Kuhn, M. & Schindelin, H. Structure of the ubiquitin-activating enzyme loaded with two ubiquitin molecules. *Acta Crystallogr Sect D Biological Crystallogr* **70**, 1311–1320 (2014).

3. Walden, H. *et al.* The Structure of the APPBP1-UBA3-NEDD8-ATP Complex Reveals the Basis for Selective Ubiquitin-like Protein Activation by an E1. *Mol Cell* **12**, 1427–1437 (2003).

4. Lois, L. M. & Lima, C. D. Structures of the SUMO E1 provide mechanistic insights into SUMO activation and E2 recruitment to E1. *Embo J* **24**, 439–451 (2005).

5. Truongvan, N., Li, S., Misra, M., Kuhn, M. & Schindelin, H. Structures of UBA6 explain its dual specificity for ubiquitin and FAT10. *Nat Commun* **13**, 4789 (2022).

6. Sauvé, V. *et al.* Mechanism of parkin activation by phosphorylation. *Nat Struct Mol Biol* **25**, 623–630 (2018).

7. Olsen, S. K. & Lima, C. D. Structure of a Ubiquitin E1-E2 Complex: Insights to E1-E2 Thioester Transfer. *Mol Cell* **49**, 884–896 (2013).

8. Williams, K. M. *et al.* Structural insights into E1 recognition and the ubiquitin-conjugating activity of the E2 enzyme Cdc34. *Nat Commun* **10**, 3296 (2019).

9. Notredame, C., Higgins, D. G. & Heringa, J. T-coffee: a novel method for fast and accurate multiple sequence alignment11Edited by J. Thornton. *J Mol Biol* **302**, 205–217 (2000).

10. Robert, X. & Gouet, P. Deciphering key features in protein structures with the new ENDscript server. *Nucleic Acids Res* **42**, W320–W324 (2014).

11. Mizushima, T. *et al.* Crystal Structure of UbcH8. (2005) doi:https://doi.org/10.2210/pdb1wzv/pdb.
