# Supplementary material for: Insights into the ISG15 transfer cascade by the UBE1L activating enzyme
Source: Nat Commun. 2023 Dec 2;14:7970. doi: 10.1038/s41467-023-43711-3 (PMC10693564; doi:10.1038/s41467-023-43711-3)
Supplement: Supplementary file 3 — Reporting Summary [file 41467_2023_43711_MOESM3_ESM.pdf]

nature portfolio

Corresponding author(s): Kirby Swatek

Last updated by author(s): 2023/11/01

Reporting Summary

Nature Portfolio wishes to improve the reproducibility of the work that we publish. This form provides structure for consistency and transparency in reporting. For further information on Nature Portfolio policies, see our Editorial Policies and the Editorial Policy Checklist.

Statistics

For all statistical analyses, confirm that the following items are present in the figure legend, table legend, main text, or Methods section.

n/a	Confirmed	
☐	☒	The exact sample size (*n*) for each experimental group/condition, given as a discrete number and unit of measurement
☐	☒	A statement on whether measurements were taken from distinct samples or whether the same sample was measured repeatedly
☒	☐	The statistical test(s) used AND whether they are one- or two-sided<br>*Only common tests should be described solely by name; describe more complex techniques in the Methods section.*
☒	☐	A description of all covariates tested
☒	☐	A description of any assumptions or corrections, such as tests of normality and adjustment for multiple comparisons
☐	☒	A full description of the statistical parameters including central tendency (e.g. means) or other basic estimates (e.g. regression coefficient) AND variation (e.g. standard deviation) or associated estimates of uncertainty (e.g. confidence intervals)
☒	☐	For null hypothesis testing, the test statistic (e.g. *F*, *t*, *r*) with confidence intervals, effect sizes, degrees of freedom and *P* value noted<br>*Give P values as exact values whenever suitable.*
☒	☐	For Bayesian analysis, information on the choice of priors and Markov chain Monte Carlo settings
☒	☐	For hierarchical and complex designs, identification of the appropriate level for tests and full reporting of outcomes
☒	☐	Estimates of effect sizes (e.g. Cohen's *d*, Pearson's *r*), indicating how they were calculated

Our web collection on statistics for biologists contains articles on many of the points above.

Software and code

Policy information about availability of computer code

Data collection	Gel imaging: Amersham Imager 600, Amersham Typhoon, Bio-Rad ChemiDoc MP imaging system; Fluorescence polarization: BMG Lab Tech SMART Control Software; Cryo-EM: Serial EM.
Data analysis	Fluorescence densitometry: ImageQuant v5.2, GraphPad Prism v9.3.1; Fluorescence polarization: R studio v4.2.0; Cryo-EM: RELION 3.1, Gautomatch v0.56, CTFFIND v4.1.9; Structural Visualization: Chimera v1.16, PyMOL v2.5.2, ChimeraX v1.3; Model Building: COOT v0.89, Phenix.refine v1.15.2.

For manuscripts utilizing custom algorithms or software that are central to the research but not yet described in published literature, software must be made available to editors and reviewers. We strongly encourage code deposition in a community repository (e.g. GitHub). See the Nature Portfolio guidelines for submitting code & software for further information.

Data

Policy information about availability of data

All manuscripts must include a data availability statement. This statement should provide the following information, where applicable:

- Accession codes, unique identifiers, or web links for publicly available datasets
- A description of any restrictions on data availability
- For clinical datasets or third party data, please ensure that the statement adheres to our policy

Data Availability Statement

Structural data will be made publicly available upon publication of the manuscript. Cryo-EM maps have been deposited to the Electron Microscopy Data Bank (EMDB) as UBE1L~UBE2L6(C98S/C102S) ISG15(C78S) accession numbers EMDB-16891 [https://www.ebi.ac.uk/pdbe/entry/emdb/EMD-16891] and EMDB-18589 [https://www.ebi.ac.uk/pdbe/entry/emdb/EMD-18589] and atomic coordinates will be made available through the Research Collaboratory of Structural Bioinformatics Protein Data Bank (RCSB PDB) as accession code 8OIF [https://doi.org/10.2210/pdb8OIF/pdb] . Published PDB accession codes used for analysis are available under the following accession codes: 6FFA [https://doi.org/10.2210/pdb6FFA/pdb], 1Z2M [https://doi.org/10.2210/pdb1Z2M/pdb], 1WZV [https://doi.org/10.2210/pdb1WZV/pdb], 3SDL [https://doi.org/10.2210/pdb3SDL/pdb], 6YVA [https://doi.org/10.2210/pdb6YVA/pdb], 4NNJ [https://doi.org/10.2210/pdb4NNJ/pdb], 1R4N [https://doi.org/10.2210/pdb1R4N/pdb], 1Y8R [https://doi.org/10.2210/pdb1Y8R/pdb], 7PYV [https://doi.org/10.2210/pdb7PYV/pdb], 6DJX [https://doi.org/10.2210/pdb6DJX/pdb], 4II2 [https://doi.org/10.2210/pdb4II2/pdb], 6NYA [https://doi.org/10.2210/pdb6NYA/pdb]. Raw uncropped images for all SDS-PAGE gels and western blots are in the source data section. Source data are provided with this paper. The authors declare there are no restrictions on the data availability of the research presented within this study and all material will be available upon request from the corresponding authors.

Research involving human participants, their data, or biological material

Policy information about studies with human participants or human data. See also policy information about sex, gender (identity/presentation), and sexual orientation and race, ethnicity and racism.

Reporting on sex and gender	not applicable
Reporting on race, ethnicity, or other socially relevant groupings	not applicable
Population characteristics	not applicable
Recruitment	not applicable
Ethics oversight	not applicable

Note that full information on the approval of the study protocol must also be provided in the manuscript.

Field-specific reporting

Please select the one below that is the best fit for your research. If you are not sure, read the appropriate sections before making your selection.

☒ Life sciences ☐ Behavioural & social sciences ☐ Ecological, evolutionary & environmental sciences

For a reference copy of the document with all sections, see nature.com/documents/nr-reporting-summary-flat.pdf

Life sciences study design

All studies must disclose on these points even when the disclosure is negative.

Sample size	Sample size calculations were not required for the experiments presented in the study. For each experiment, the sample size was chosen to ensure reproducibility and facilitate accurate interpretation of the results. The rationale for sample size was based on previous experience with these and related assays and is in line with the literature for similar studies.
Data exclusions	No data were excluded.
Replication	All experiments were performed independently in at least duplication, but most were performed in triplicate. All attempts at replication were successful.
Randomization	Randomization was not required due to the small number of samples per experiment and the experimental controls throughout the study.
Blinding	Blinding was not required due to the small number of samples per experiment and the experimental controls throughout the study.

Reporting for specific materials, systems and methods

We require information from authors about some types of materials, experimental systems and methods used in many studies. Here, indicate whether each material, system or method listed is relevant to your study. If you are not sure if a list item applies to your research, read the appropriate section before selecting a response.

Materials & experimental systems

n/a	Involved in the study
☐	☒ Antibodies
☐	☒ Eukaryotic cell lines
☒	☐ Palaeontology and archaeology
☒	☐ Animals and other organisms
☒	☐ Clinical data
☒	☐ Dual use research of concern
☒	☐ Plants

Methods

n/a	Involved in the study
☒	☐ ChIP-seq
☒	☐ Flow cytometry
☒	☐ MRI-based neuroimaging

Antibodies

Antibodies used

Monoclonal anti-FLAG (Merck, Cat. no. F3165, clone name M2, 1:1,000 dilution for Western blots), Monoclonal anti-ubiquitin (Novus, Cat. no. NB300-130, clone name Ubi-1, 1:1,000 dilution for Western blots), Monoclonal anti-beta actin (ProteinTech, Cat. no. 66009, clone name 2D4H5, 1:5,000 dilution for Western blots), Monoclonal anti-UBE1L (Santacruz, Cat. no. sc-390097, clone name B-7, 1:1,000 dilution for Western blots), Monoclonal anti-HA tag (abcam, Cat. no. ab18181, clone name HA.C5, 1:1,000 dilution for Western blots), Polyclonal anti-HERC5 (ThermoFisher, Cat. no. PA5-100555, 1:1,000 dilution for Western blots), Polyclonal anti-mouse secondary HRP (ThermoFisher, Cat. no. 31450, 1:5,000 dilution for Western blots), Polyclonal anti-rabbit secondary HRP (ThermoFisher, Cat. no. 31460, 1:5,000 dilution for Western blots).

Validation

The manufacturers validated all antibodies for western blotting. Please see the manufacturers' websites for further information. The antibodies were further validated in this study (see Figs. 6a,b and Supplementary Fig. 9)

Eukaryotic cell lines

Policy information about cell lines and Sex and Gender in Research

Cell line source(s)

HeLa cells (CCL-2) were obtained from ATCC, Sf9 cells were obtained from Thermo Fisher (Cat. no. 11496015), and High Five cells were obtained from Thermo Fisher (Cat. no. B85502).

Authentication

Cell lines displayed normal cell morphology and were not further authenticated.

Mycoplasma contamination

HeLa, Sf9, and Hi5 cells were routinely check for mycoplasma contamination and were negative.

Commonly misidentified lines
(See ICLAC register)

No commonly misidentified cell lines were used in this study.

Plants

Seed stocks

not applicable

Novel plant genotypes

not applicable

Authentication

not applicable
